# An innovative cell-based transplantation therapy for an immature permanent tooth in an adult: a case report

**DOI:** 10.1186/s12903-024-04410-7

**Published:** 2024-06-01

**Authors:** Keyue Liu, Wenxu Li, Sijing Yu, Guimin Li, Ling Ye, Bo Gao

**Affiliations:** State Key Laboratory of Oral Diseases, National Clinical Research Center for Oral Diseases, Dentistry and Endodontics Department, West China Hospital of Stomatology, Sichuan University, Chengdu, China

**Keywords:** Case report, Regenerative endodontic procedure, Cell-based transplantation therapy, Autologous human dental pulp cells, Liquid phase concentrated growth factor

## Abstract

**Background:**

Immature teeth with necrotic pulps present multiple challenges to clinicians. In such cases, regenerative endodontic procedures (REPs) may be a favorable strategy. Cells, biomaterial scaffolds, and signaling molecules are three key elements of REPs. Autologous human dental pulp cells (hDPCs) play an important role in pulp regeneration. In addition, autologous platelet concentrates (APCs) have recently been demonstrated as effective biomaterial scaffolds in regenerative dentistry, whereas the latest generation of APCs—concentrated growth factor (CGF), especially liquid phase CGF (LPCGF)—has rarely been reported in REPs.

**Case presentation:**

A 31-year-old woman presented to our clinic with the chief complaint of occlusion discomfort in the left mandibular posterior region for the past 5 years. Tooth #35 showed no pulp vitality and had a periodontal lesion, and radiographic examination revealed that the tooth exhibited extensive periapical radiolucency with an immature apex and thin dentin walls. REP was implemented via transplantation of autologous hDPCs with the aid of LPCGF. The periodontal lesion was managed with simultaneous periodontal surgery. After the treatment, the tooth was free of any clinical symptoms and showed positive results in thermal and electric pulp tests at 6- and 12-month follow-ups. At 12-month follow-up, radiographic evidence and three-dimensional models, which were reconstructed using Mimics software based on cone-beam computed tomography, synergistically confirmed bone augmentation and continued root development, indicating complete disappearance of the periapical radiolucency, slight lengthening of the root, evident thickening of the canal walls, and closure of the apex.

**Conclusion:**

hDPCs combined with LPCGF represents an innovative and effective strategy for cell-based regenerative endodontics.

## Introduction

Trauma, deep caries, and developmental dental anomalies such as dens evaginatus (DE) are common etiologies of pulp necrosis in immature teeth [1], leading to thin dentine walls, immature open apex, and periapical lesions [2]. In such cases, traditional root canal therapy or apexification technique may not achieve satisfactory outcomes because of dental pulp loss and cessation of root development [3]. Therefore, to promote continued root development and restore pulp vitality, regenerative endodontic procedures (REPs) may be a promising alternative treatment for immature teeth with pulp necrosis/apical periodontitis [4].

REPs are biologically based procedures designed to replace damaged structures, including dentin and root structures, as well as cells of the pulp–dentin complex [5]. Compared with apical plug technique, REPs have been reported to allow the continued development of roots in immature teeth with necrotic pulps [6]. Two strategies in REPs have been applied, including cell homing and cell-based therapy [7]. In cell homing therapy, regeneration is accomplished by promoting de novo host stem cell tissue formation, also known as cell-free therapy [8]. Cell-based therapy involves the transplantation of autologous or allogeneic stem/progenitor cells [9]. In practice, cell homing therapy may be simpler and more economical to perform than cell-based therapy [7]. For example, induced bleeding by over-instrumentation is commonly suggested in cell homing therapy [10]. Besides, using growth factors that promote chemotaxis and proliferation/differentiation of stem cells can potentially facilitate clinical success [11]. However, the ability of the cells to migrate can limit the success of the cell homing strategy [12]. In adult patients, the microenvironment of the canal space, vitality of Hertwig’s epithelial root sheath, and apical papilla may vary significantly compared with those of younger individuals [7]. Therefore, in adult patients, in whom the circulating stem cell concentration may be lower in both number and quality, a cell-based procedure may be required to ensure success [13].

Recently, the transplantation of autologous dental pulp cells with the aid of biomaterial scaffolds or signaling molecules for pulp regeneration has attracted attention because of its promising outcomes [9]. Transplantation of autologous dental pulp cells under strict good manufacturing practice (GMP) standards has preserved many nonvital teeth, whether they are mature [14–16] or immature [17], single-rooted [14, 15, 17] or multi-rooted [16]. As a scaffold abundant with necessary growth factors, autologous platelet concentrates (APCs), including platelet rich plasma (PRP), platelet rich fibrin (PRF), concentrated growth factor (CGF), and liquid phase CGF (LPCGF), are effective in regenerative dentistry. They have contributed to the successful treatment of immature permanent teeth [18] and the healing of bone defects [19]. Based on its liquid property, LPCGF has been developed into an injectable filler, which could render biomaterial scaffold more practical for successful pulp regeneration therapy [9]. To the best of our knowledge, there are no reports describing the appliance of LPCGF in regenerative endodontics. Therefore, we aim to first report a cell-based REP to treat an immature tooth in an adult by transplanting autologous human dental pulp cells (hDPCs) with the aid of LPCGF.

## Case presentation

**Fig. 1:**
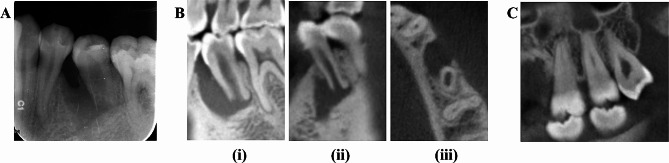
(**A**) The preoperative IOPA radiography of tooth #35, depicting thin dentine walls and an open apex, with extensive periapical radiolucency. (**B**) The preoperative CBCT images from sagittal (i), coronal (ii) and horizontal (iii) planes, further revealed the extent of the bony defect and the large diameter of the immature apex. (**C**) CBCT information of the third molar tooth #28 in the sagittal view, showing no dentinal and periapical radiolucency

A 31-year-old woman presented to our clinic with the chief complaint of occlusion discomfort in the left mandibular posterior region for the past 5 years. During the course of disease, self-subsided gingival pustules occurred several times; however, no history of spontaneous or temperature-provoked pain was reported. The patient’s medical, dental, and family history did not contribute to her condition. Extraoral examinations revealed normal findings. Intraoral examinations revealed no caries, but a fractured DE was observed in the left second mandibular premolar (tooth #35). Mucosal swelling and the sinus tract were visible in the buccal region. It was tender to percussion and periapical palpation. Thermal vitality tests (1,1,1,2-tetrafluoroethane and hot gutta-percha bar) and electric pulp test (Pulp Tester, Denjoy, China) showed negative results. In addition, the periodontal examination revealed the presence of grade “I” mobility and deep periodontal pockets with probing depths of > 5 mm. An intraoral periapical (IOPA) (Carestream Health, USA) examination revealed large periapical radiolucency, thin dentine walls, and an open apex. Cone-beam computed tomography (CBCT) (J. MORITA MFG. CORP, Japan) images further confirmed the extent of the bony defect and the large diameter of the immature apex (Fig. 1A, B). Clinically and radiographically, tooth #28 had no dentine or periapical lesions, and it had no occlusal relationship with the mandible teeth; therefore, tooth #28 was recommended for extraction (Fig. 1C). The patient was diagnosed with DE, combined pulpal–periodontal lesion, pulp necrosis, and symptomatic apical periodontitis in tooth #35. Treatment procedures, including nonsurgical root canal with an apical plug, extraction, and REP, were provided. The patient was informed that the goal of REP was to restore pulp vitality and initiate continued root development, which may not be successful. A decision was made to perform REP via the transplantation of autologous hDPCs with LPCGF. Simultaneous periodontal surgery was also considered for the management of periodontal lesion. Written informed consent was obtained, and the patient was scheduled for treatment. Consensus-based Clinical Case Reporting Guidelines (CARE) were used as standard guidance to report the case flow and process of treatment [20].

## First treatment visit

The oral cavity was rinsed with 5 ml of 0.12% chlorhexidine for 1 min. Tooth #35 was isolated using a rubber dam (OptiDam, Kavo Kerr SybronEndo, USA) under superficial anesthesia with tetracaine hydrochloride. The working length, diameter of the apical apex, and position of the cementoenamel junction (CEJ) were estimated radiographically. The canal was accessed and gently irrigated with copious amounts of 1% sodium hypochlorite, followed by sterile saline solution using a side-vent syringe (NaviTips, Ultradent, USA) and an ultrasonic irrigator tip (ACTEON, SATELEC, France). After drying with sterile paper points, the root canal was dressed with calcium hydroxide paste (ApexCal, Ivoclar Vivadent, Schaan, Liechtenstein). A sterile cotton pellet was placed, and the access cavity was sealed with 3–4-mm Caviton (Fuji GC, Japan). Tooth #35 was made occlusal adjustment to make sure there was no trauma from occlusion.

## Second treatment visit

**Fig. 2:**
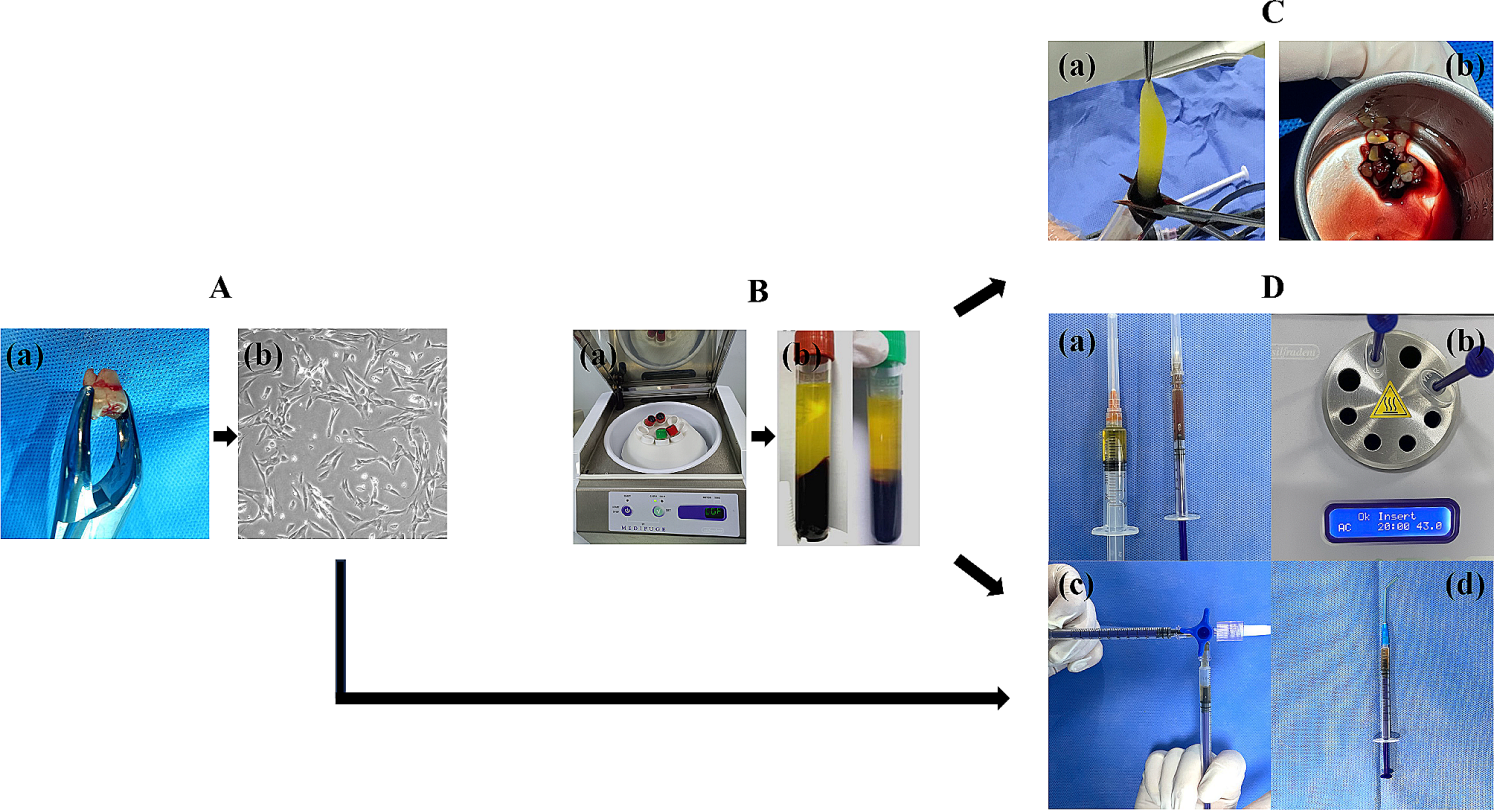
(**A**) The third molar tooth #28 was extracted (**a**) and initiated cell isolation and cultivation (**b**). (**B**) Blood centrifugation with Medifuge (**a**). CGF was prepared with a red-cap tube; LPCGF was prepared with a green-cap tube (**b**). (**C**) Gel CGF was separated (**a**) and cut into fragments for periodontal surgery (**b**). (**D**) (**a**) In LPCGF system, 0.5 ml of CD34^+^ stem cell layer was collected in a 1-ml syringe (right), 2 ml of LPCGF was collected with a 5-ml syringe (left). (**b**) The mixture of LPCGF (2 ml) and CD34^+^ stem cells (0.5 ml) was transferred into APAG device and treated at 43 °C for 20 min. (**c**) After cooling down to room temperature, the released 1 × 10^7^ hDPCs in 200 µl of thermally-treated LPCGF (vertical) was infiltrated into another 200 µl of treated-LPCGF (horizontal) with a female-female Luer lock connector for at least 60s. (**d**) The injectable hDPCs/LPCGF complex was prepared for regenerative endodontic treatment

The intracanal medicament was replaced after 2 weeks because tooth #35 was still slightly tender to percussion. Simultaneously, the third molar tooth #28 was extracted under local anesthesia using Primacaine (4% articaine, 1:100000 epinephrine) (Fig. 2A (a)). The extracted tooth was briefly stored in a sterile test tube (15 ml) containing 9 ml of 1× phosphate buffered saline (1× PBS) (Gibco, USA) and 1 ml of penicillin/streptomycin (Gibco, USA), and the tooth was immediately transported on ice within 1 h to initiate cell culture (Fig. 2A (b)). The sample was manipulated aseptically under a laminar flow hood. After washing thrice with 1× PBS (Gibco, USA) containing 1% penicillin/streptomycin (Gibco, USA), the cleaned tooth was separated longitudinally to acquire the pulp tissue using micro-tweezers. The hDPCs were isolated and cultured as previously described with slight modifications [21, 22]. Briefly, the separated pulp tissue underwent an enzymatic digestion reaction with 3 mg/ml collagenase type I solution (Biofroxx, Germany) for 1 h in a cell incubator. The solution was filtered through a 70-µm strainer (BD Falcon, USA), and the single cell suspension was placed in a 25-cm^2^ culture flask containing moderate complete medium (1× DMEM [Gibco] + 10% human AB serum [Sigma–Aldrich] + 1% penicillin/streptomycin [Gibco]). The culture flask was incubated at 37 °C under humidified air and 5% CO_2_. Upon reaching 70–80% confluency, the cells were harvested using 0.25% trypsin/EDTA (Gibco, USA). For clinical use, 1 × 10^7^ hDPCs at passage 3 were released without contamination and washed thrice with physiological saline before transplanting them into the root canal.

## Third treatment visit

The patient returned at 5-week follow-up. The buccal swelling subsided and the sinus drainage healed. There was no percussion or palpation tenderness; however, grade “I” periodontal mobility and deep probing depths (> 5 mm) still existed.

The first step was the preparation of CGF and LPCGF. Autologous peripheral blood was collected via antecubital venipuncture in centrifuge tubes (one tube with a red cap and one tube with a green cap) (Vacuette, 9 ml, Greiner Bio-One, Austria). Blood samples were collected and centrifuged in a specific centrifugal accelerator (Medifuge, Silfradent, Italy) according to Sacco’s protocol (Fig. 2B (a)) as follows: acceleration for 30 s, 2700 rpm for 2 min, 2400 rpm for 4 min, 2700 rpm for 4 min, 3000 rpm for 3 min, deceleration for 36 s, and termination [23]. At the end of the process, CGF in the red-cap tube and LPCGF in the green-cap tube were separated from the upper liquid platelet poor plasma (PPP) layer and the lower red blood cell (RBC) layer (Fig. 2B (b)). Between the LPCGF and RBC layers, there was approximately 0.5 ml of liquid with abundant CD34^+^ stem cells, which was designated the CD34^+^ stem cell layer [24]. For subsequent periodontal surgery, the CGF clot was separated in the middle of the red-cap tube and was carefully cut into small pieces with sterile scissors (Fig. 2C (a-b)). LPCGF was prepared according to the manufacturer’s instructions. The CD34^+^ stem cell layer (0.5 ml) and LPCGF (2 ml) were collected from the green-cap tube and thoroughly remixed using a female–female Luer lock connecter. The mixture was thermally treated using Activated Plasma Albumin Gel (APAG) device (Silfradent, Italy) at 43 °C for 20 min (Fig. 2D (a-b)). After cooling to room temperature, 1 × 10^7^ hDPCs in 200 µl of thermally treated LPCGF were introduced into equivalent treated-LPCGF through another Luer lock connector for at least 60 s (Fig. 2D (c)) to prepare the homogeneous hDPC/LPCGF complex (Fig. 2D (d)).

**Fig. 3:**
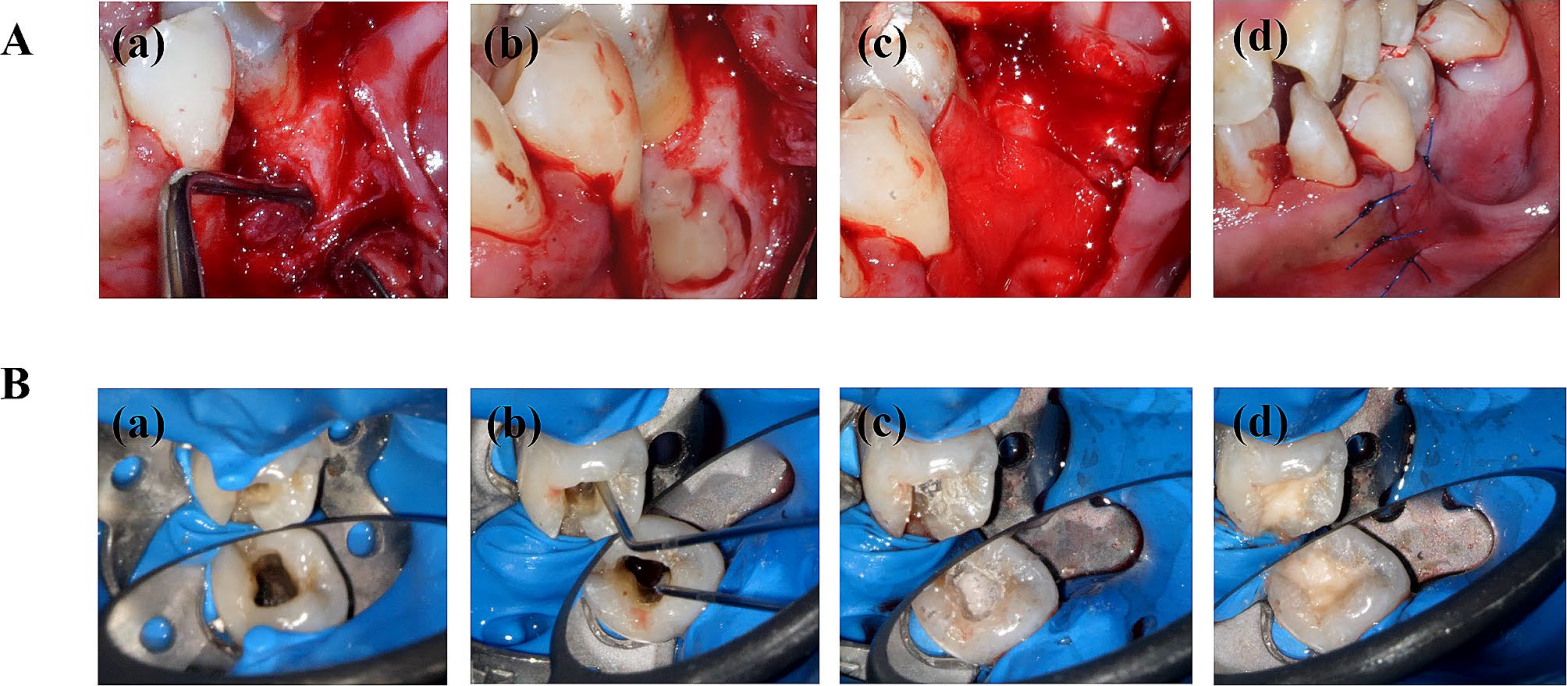
(**A**) The process of periodontal surgery. (**a**) Diseased soft tissue was debrided by curettage. (**b**) CGF was grafted into the defect region. (**c**) Collagen membrane was placed over the grafted defect. (**d**) Flap was repositioned and wound was closured with sutures. (**B**) The process of REP. Tooth #35 was locally anesthetized and under rubber dam isolation. (**a**) Pulp cavity was accessed, the canal was subsequently irrigated and dried. (**b**) Prepared hDPCs/LPCGF complex was injected into the canal up to approximately 3 mm below the CEJ. (**c**, **d**) Access was carefully sealed with 3-mm iRoot BP plus paste and glass ionomer cement

**Fig. 4:**
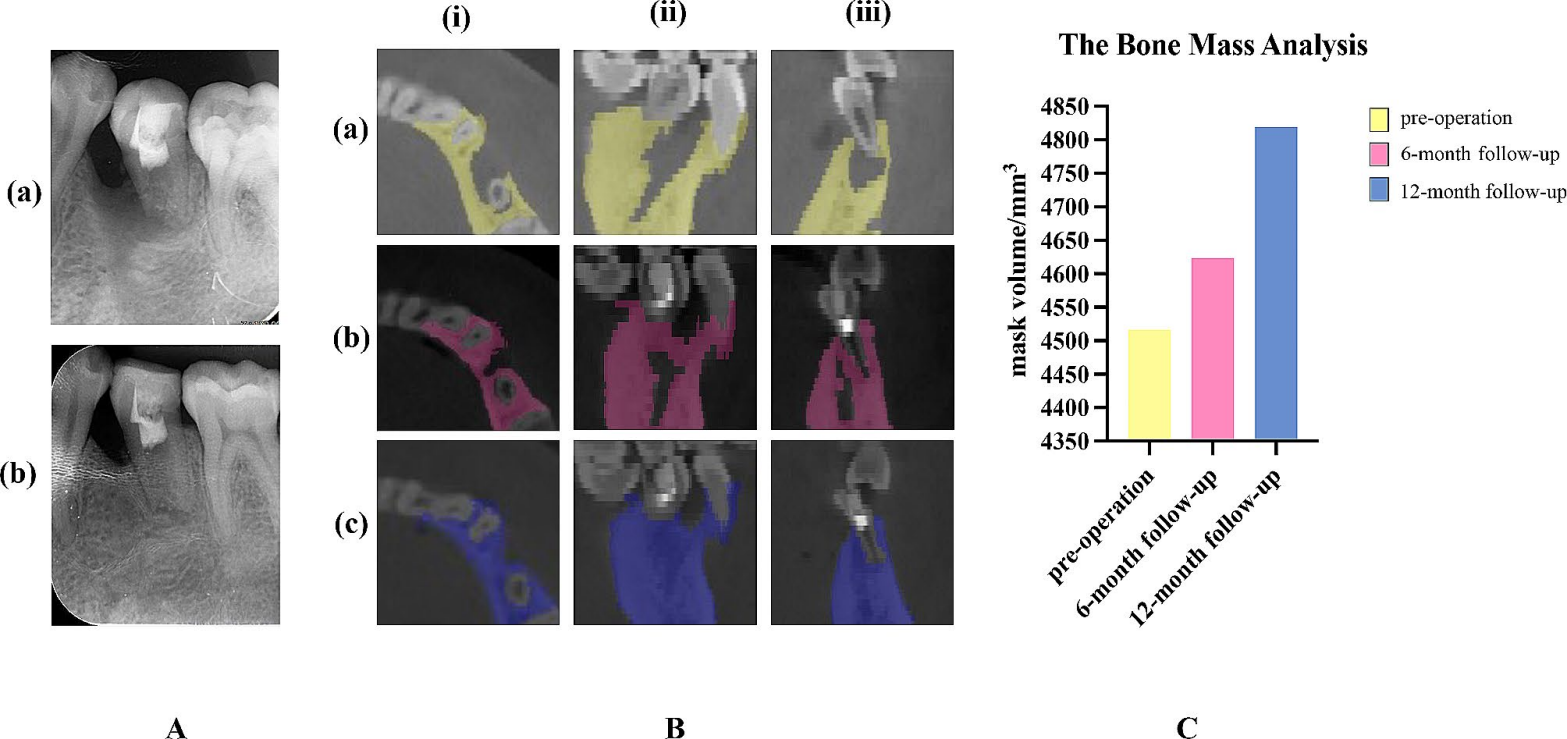
(**A**) IOPA radiographies after treatments. (**a**) Immediate postoperative IOPA radiography confirmed the filling quality. (**b**) Postoperative IOPA radiography at 12-month follow-up indicated complete regression of periapical radiolucency. (**B**) CBCT images analyzed using Mimics software. The pre-operation (**a**), 6-month follow-up (**b**), and 12-month follow-up (**c**) CBCT images (at 90 kV, 10 mA, and a scanning time of 32 s) (J. MORITA MFG. CORP, Japan) revealed the resolution of periapical radiolucency from horizontal (i), sagittal (ii), and coronal (iii) planes. The periapical radiolucency was completely disappeared at 12-month follow-up. (**C**) The analysis of bone mass in a fixed range from tooth #33 to tooth# 36 (Mimics Medical 17.0). The increasing mask volumes suggested bone mass augmentation process (yellow mask volume for pre-operation = 4515.5625 mm^3^; red mask volume for 6-month follow-up = 4623.3184 mm^3^; blue mask volume for 12-month follow-up = 4818.9810 mm^3^)

The second step involved surgical enucleation of the periodontal lesion. Local block and infiltration anesthesia was administered with 1% lidocaine containing vasoconstrictor. A full-thickness flap was raised using intrasulcular and vertical incisions. A mass of the diseased soft tissue was exposed and removed via curettage (Fig. 3A (a)). After defect debridement, the CGF fragments were grafted into the defect region (Fig. 3A (b)). Subsequently, a collagen membrane (Bio-Guide, Geistlich Biomaterials, 13*25 mm, Switzerland) was applied over the grafted defect (Fig. 3A (c)). The flap was repositioned coronally and stabilized without tension. Hemostasis was achieved, and wound closure was performed using 5–0 nonresorbable sutures (PROLENE, ETHICON, USA) (Fig. 3A (d)). A few minutes after suturing, tooth #35 that was isolated using a rubber dam was accessed, the calcium hydroxide paste was flushed with copious amounts of saline, and the canal was irrigated with 1% sodium hypochlorite (20 ml/canal). The residue of the irrigation solution was thoroughly washed with saline and 17% EDTA for 5 min for dentin conditioning. After rinsing with sterilized saline, the canal was dried with paper points (Fig. 3B (a)). During root canal irrigation and drying, the hDPCs/LPCGF complex was prepared, and ~ 20 µl of the complex containing 5 × 10^5^ hDPCs was injected into the canal up to approximately 3 mm below the CEJ with the aid of a surgical microscope (OPMI pico, Carl Zeiss AG, Germany) (Fig. 3B (b)). After separation using an absorbable gelatin sponge (Xiang’en, China), 3-mm iRoot BP plus paste (Innovative BioCeramix, Canada) and glass ionomer cement (Fuji IX GP, Japan) were applied to close the access (Fig. 3B (c-d)). Immediate postoperative radiography confirmed the filling quality (Fig. 4A (a)). Postoperatively, pain control was achieved via ibuprofen administration (300 mg, twice daily for 3 days). The patient was advised to use 0.12% chlorhexidine mouthwash twice daily for 1 week. The sutures were removed after 7 days, and the tooth was permanently restored with composite resin (3 M, ESPE, USA).

## Follow-up examination

**Fig. 5:**
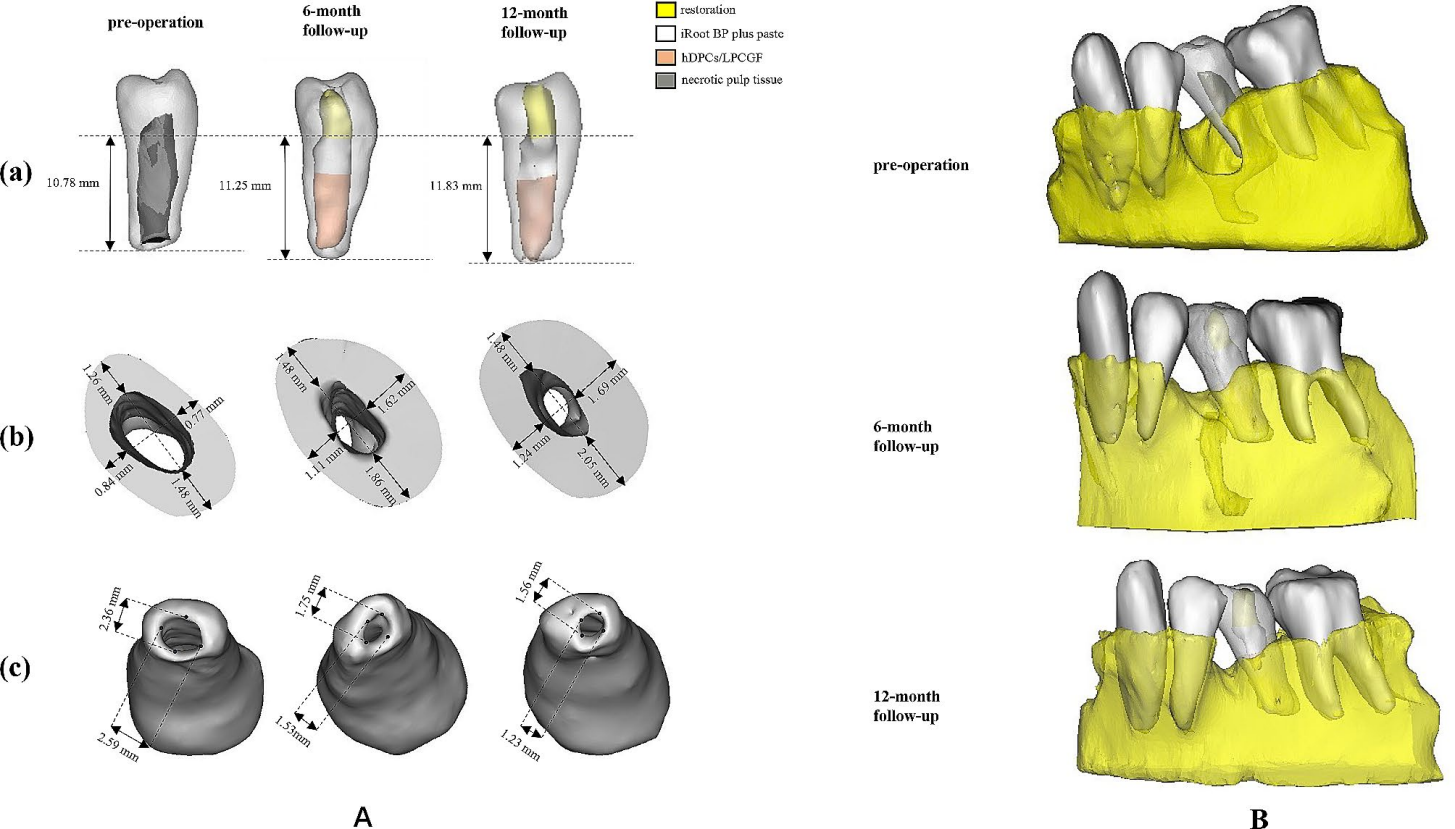
(**A**)3D-reconstructed models of tooth #35 at pre-operation, 6-month follow-up, and 12-month follow-up (Mimics Medical 17.0). The simulated images showed continued root development. (**a**) Lengthening of the root. The measurements of root length slightly increased: 10.78 mm at pre-operation; 11.25 mm at 6-month follow-up; 11.83 mm at 12-month follow-up. (**b**) Thickening of the canal walls (the measured layer was at 3 mm below the CEJ). The measurements of the thickness of four representative dentine walls increased: 1.26 mm*0.77 mm*1.48 mm*0.84 mm at pre-operation; 1.48 mm*1.62 mm*1.86 mm*1.11 mm at 6-month follow-up; 1.48 mm*1.69 mm*2.05 mm*1.24 mm at 12-month follow-up). (**c**) Closure of the apex. The measurements of apical diameter decreased: 2.36 mm*2.59 mm at pre-operation; 1.75 mm*1.53 mm at 6-month follow-up; 1.56 mm*1.23 mm at 12-month follow-up. (**B**) In addition, the simulated images made an apparent exhibition of the bone healing process from pre-operation to 12-month follow-up

**Fig. 6:**
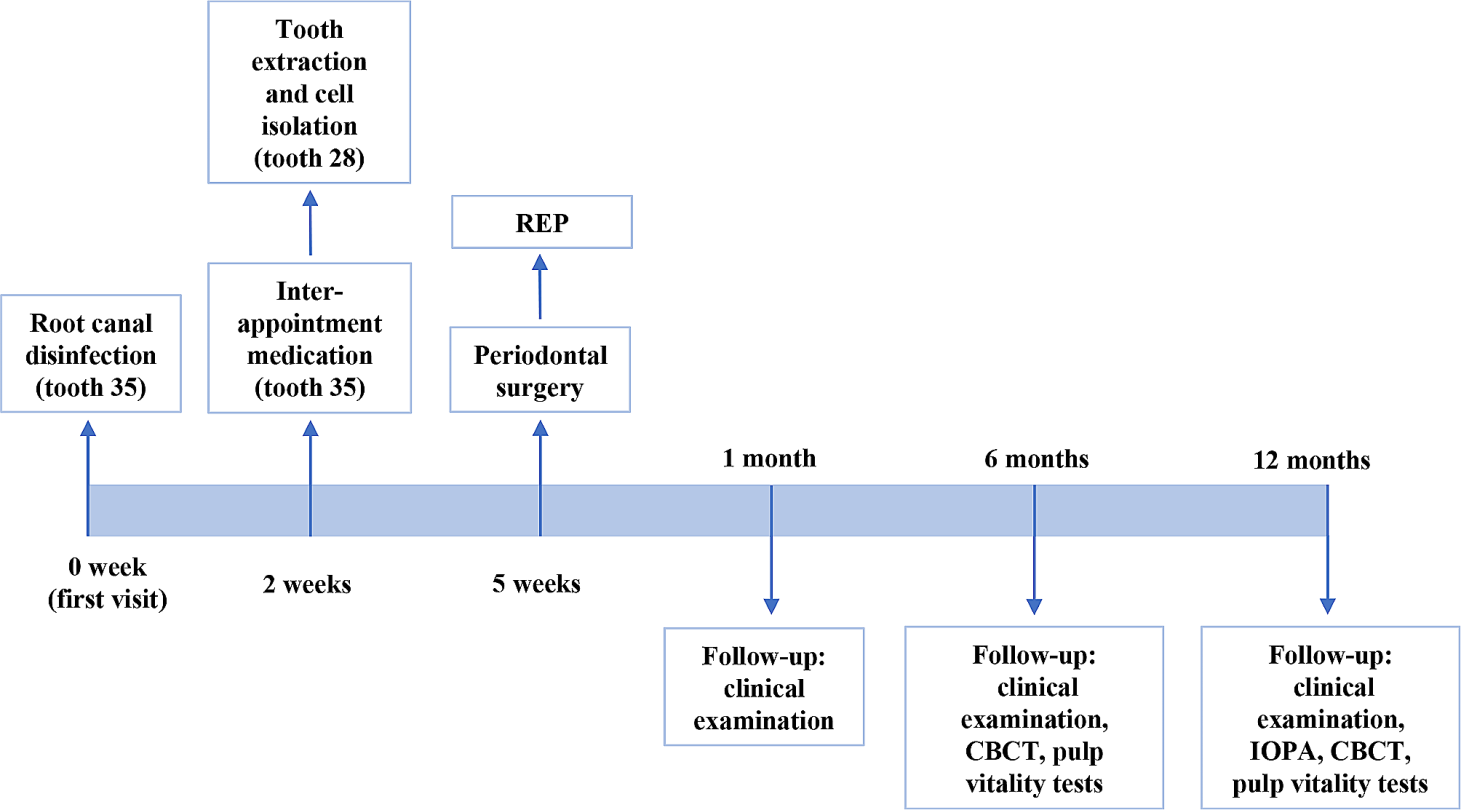
Timeline of the treating procedure. At the first visit (0 week), tooth #35 was accessed and root canal was disinfected with irrigation and intra-canal medication. At the second visit (2 weeks), intra-canal medicament was replaced because of tenderness to percussion and palpation. Tooth #28 was extracted to initiate cell culture. At the third visit (5 weeks), dental pulp cells were harvested and CGF/LPCGF was prepared for simultaneous periodontal surgery and REP treatment. One month after treatment, patient was received clinical examination. At 6-month follow up and 12-month follow up, patient was re-examined, and the treatment outcomes were re-valued by radiographic examinations (IOPA and/or CBCT) and pulp vitality tests (thermal and electrical)

The patient was kept under regular radiographic and clinical follow-ups at 1, 6, and 12 months after treatment. The patient remained free of any symptoms and was satisfied with the treatment outcome. Tooth #35 was asymptomatic of percussion and periapical palpation. Periodontal examination revealed normal physiological mobility and probing depths (< 3 mm). At the 6-month and 12-month follow-up, tooth #35 showed positive results in thermal and electric pulp tests. IOPA and CBCT images, which were evaluated by the same specialist endodontist, showed intact periapical bone structure at the last examination(Fig. 4A (b), B)The bone mass was measured using the volumes calculated by a semiautomatic segmentation process (Mimics Medical 17.0, Materialise, Leuven, Belgium) [25]. The analysis was repeated independently by three researchers. The measurements were analyzed using the ICC (Intraclass Correlation Coefficient). The analysis indicated a high degree of consistency among the researchers’ measurements. The average of the measurements was taken to represent the final results. The results showed increased mask volumes during the follow-ups, indicating bone augmentation in the bony defect after treatment (Fig. 4C). Moreover, a slight lengthening of the root, thickening of the canal walls and closure of the apex were confirmed by three-dimensional models reconstructed using the Mimics software (Fig. 5A, B). The treatment timeline is provided in Fig. 6.

## Discussion

Importantly, several issues should be taken into consideration before conducting REPs, such as the patients’ age and periodontal condition [26]. REP using blood induction technique is often implemented in age ranging from 9 to 18 years old, but younger patients (9 to 13 years old) can be better candidates attributed to a greater healing capacity [10]. In our case, the transplantation of autologous hDPCs with LPCGF may have contributed to the successful results because of the lower regenerative potential associated with the patient’s age [27]. Besides, REPs are not applicable in teeth with combined pulpal–periodontal lesion [10]. In our case, we have medicated in root canal for 5 weeks, while the deep periodontal pocket still existed. And we didn’t think about prolonging the duration of intra-canal medication, for the risk of root fracture due to the long-term use of calcium hydroxide medicament [28]. Therefore, we were more inclined to conduct simultaneous periodontal surgery for the success of the cell transplantation therapy.

hDPCs contain a heterogeneous mesenchymal stem cell population. Depending on their multipotency and sensitivity to local paracrine activity, hDPCs can migrate, proliferate, and release angiogenic factors and neurogenic factors, facilitating angiogenesis and neurogenesis as well as pulp-dentin complex regeneration [29]. Stem cells from bone marrow (BMSCs) and adipose tissue (ADSCs) have pulp regenerative potential as well, but their regenerative capacity for pulp tissues, angiogenesis, and reinnervation was weaker than that of stem cells from dental pulp. And hDPCs are isolated relatively easy from extracted third molars with no risk to the donor [30]. The autologous hDPCs transplantation technique presents a new perspective to cell-based pulp regeneration [17]. In previous clinical trials, autologous hDPCs have been isolated and cultured under strict GMP standards. The ex vivo expanded hDPCs were de novo transplanted for regenerative endodontic therapy [15, 16]. In Xuan’s study, autologous deciduous pulp cells were isolated and fabricated into three-dimensional cell aggregates. The harvested cell aggregates were implanted into the root canal and packed into the apical end of the root. After 12 months, continued root development and restored pulp sensitivity were observed [17]. Nakashima et al. developed modified dental pulp cells to facilitate the regeneration process [15, 16]. By exposing dental pulp cells to granulocyte-colony stimulating factor (G-CSF) or hypoxia environment, the stemness and regenerative potential of the preconditioned dental pulp cells were enhanced [31, 32]. Under excellent quality control, the transplantation of G-CSF-mobilized dental pulp stem cells (MDPSCs) or hypoxia-preconditioned dental pulp stem cells (hpDPSCs) in affected teeth provided successful treatment outcomes. Clinical and radiographic evidence indicated the resolution of symptoms and remodeling of the roots. The responsive results of pulp vitality tests and high-signal intensity in magnetic resonance imaging (MRI) revealed pulp regeneration in the treated teeth [15, 16].

Optimized cell delivery systems are key to successful regenerative therapy [13]. Biomaterial scaffolds with suitable growth factors can provide a three-dimensional space for the growth and metabolism of stem cells, thus accelerating the regeneration process [30]. The biomaterials of scaffolds are classified as naturally derived or synthetically engineered polymers, ceramics, and composites [33]. Atelocollagen scaffold is one of the most commonly used collagen-derived scaffolds in cell-based pulp regeneration [9]. It can be easily introduced into root canals because of its liquid property and has successfully carried autologous hDPCs with growth factors in cell-based REP [15, 16]. A bioactive ceramic scaffold (PerioGlas®) was also effective for regenerative endodontics when combined with stem cells from human exfoliated deciduous teeth (SHEDs) [34, 35]. Some examples of natural scaffolds are host-derived materials, such as APCs, which are collected from peripheral blood and have been considered a “good healer” in regenerative dentistry [18]. PRP, also known as the first generation of platelet concentrates, provided successful clinical outcomes in revascularization of immature teeth [36, 37]. The positive ability of the second-generation PRF in regenerative endodontics has also been demonstrated in animal models and clinical studies. In vivo transplantation of autologous dental pulp cells/PRF constructs in canine models resulted in orthotopic de novo regeneration of pulp-like tissue with abundant blood capillaries and dentine deposition at 8 weeks postoperatively [38]. This innovative therapy was further designed as a personalized cell therapy for a mature permanent tooth with pulpitis. By transplanting autologous hDPCs with leukocyte-PRF (L-PRF) clots into the root, the tooth was found to be responsive to cold and electric pulp tests after the treatment. Laser Doppler Flowmetry indicated blood perfusion at 36-month follow-up. This suggests the potential use of a combination of autologous dental pulp cells and platelet concentrate scaffold in regenerative endodontics [14].

As the third generation of platelet concentrate products, CGF is prepared by centrifuging blood samples in a specific centrifugal accelerator (Medifuge, Silfradent, Italy) [23]. Compared with the previous two generations of APCs, CGF consists of a denser and stronger scaffold structure and provides a higher concentration and longer lasting release of growth factors compared with PRP or PRF [39, 40]. As a combination product of biomaterial scaffold and cytokines, CGF has great potential in the field of bone regeneration [19]. CGF promotes the proliferation and osteogenic differentiation of periodontal ligament stem cells and bone marrow cells, making it an effective adjuvant for the guided bone regeneration procedure in healing alveolar socket defects [41], periodontal bony defects [42] and chronic periapical lesions [43]. Moreover, CGF has been recognized as a positive modulator of dental pulp cells to induce the regeneration of the pulp–dentin complex [44, 45] and facilitate root development of immature teeth in clinical situations [46]. Recently, the liquid phase of CGF (known as LPCGF) has been further formulated. LPCGF is elaborated with green-cap centrifugation tubes, which enable the isolation of liquid fibrin because of the anticoagulant on the tube walls. CGF (gel phase CGF) is obtained by using red-cap tubes with textured walls, which promote fibrin polymerization process [47, 48]. By applying APAG, a low-plate plasma heating device (Silfradent, Italy), the thermally treated LPCGF can be reconstructed and become more progressive in regenerative procedures [49]. Characterized with its injectable modality and collagen regeneration ability, LPCGF has become attractive in aesthetic and cosmetology medicine [50]. In addition, LPCGF plays an important role in regenerative dentistry [51]. Recently, Yu et al. reported that LPCGF promoted the proliferation, migration, and odontogenic differentiation of hDPCs. Heterotopic transplantation of the hDPCs/LPCGF complex resulted in pulp-like tissue regeneration [52]. Therefore, it will be innovative and foresighted to develop an injectable filler by incorporating the LPCGF with hDPCs for regenerative endodontic therapy.

Although various basic and preclinical studies have been conducted to assess the efficacy and safety of cell transplantation for endodontic treatment [30, 53], cell-based procedures have many challenges, including economic and ethical concerns. These challenges include stem cell isolation, ex vivo expansion, good manufacturing practice facilities, stem cell banks, government regulatory issues, clinician skills, staff training, and a comparatively high cost [8], therefore, this technique has not been performed on a large scale. Iohara et al. found that dental pulp cells showed no contamination, no abnormalities/aberrations in karyotype, and no tumor formation after ex vivo expansion for 7 passages and still exhibited great efficacy in pulp regeneration when transplanted into a canine pulpectomized tooth [54]. A previous clinical study also demonstrated that an autologous dental pulp cell transplantation strategy for pulp regeneration had no adverse effects on systemic health during the 2-year follow-up period [17]. In our clinical case, histological examination was challenging. The newly formed tissues may be ectopic, such as soft connective tissues, cementum, or bone, instead of the true regenerated pulp [55]. However, the reported cell therapy satisfied the three outcomes expected by REPs, including resolution of clinical signs and symptoms, further maturation of the root, and neurogenesis [8]. Furthermore, no adverse events were observed after the treatment, but safe evaluation for a longer period is required [56].

## Conclusion

Herein, we proposed an innovative and effective strategy for cell-based REPs. We first combined hDPCs with LPCGF to manage an immature tooth in an adult successfully; however, the applying of LPCGF in regenerative endodontics is still at an early stage of research, additional histological experiments and high-quality clinical studies with a large sample size and longer follow-up are warranted to validate this treatment approach.

## Data Availability

All data underlying the findings and outcome are presented as part of the article and no supplementary source data are required.

## Abbreviations

DE: Dens evaginatus
REPs: Regenerative endodontic procedures
APCs: Autologous platelet concentrates
PRP: Platelet rich plasma
PRF: Platelet rich fibrin
L-PRF: Leukocyte-platelet rich fibrin
CGF: Concentrated growth factor
LPCGF: Liquid phase of concentrated growth factor
hDPCs: Human dental pulp cells
MDPSCs: Mobilized dental pulp stem cells
hpDPSCs: Hypoxia-preconditioned dental pulp stem cells
SHEDs: Stem cells from human exfoliated deciduous teeth
IOPA: Intra-oral periapical
CBCT: Cone-beam computed tomographic
MRI: Magnetic resonance imaging
CEJ: Cementoenamel junction
APAG: Activated Plasma Albumin Gel
